# Mechanical Relaxation-to-Rejuvenation Transition in a Zr-based Bulk Metallic Glass

**DOI:** 10.1038/s41598-017-00768-7

**Published:** 2017-04-04

**Authors:** M. Zhang, Y. M. Wang, F. X. Li, S. Q. Jiang, M. Z. Li, L. Liu

**Affiliations:** 1 School of Materials Science and Engineering, Huazhong University of Science and Technology, Wuhan, 430074 China; 2 State Key Laboratory of Nonlinear Mechanics, Institute of Mechanics, Chinese Academy of Sciences, Beijing, 100190 China; 3 Department of Physics, Beijing Key Laboratory of Opto-electronic Functional Materials & Micro-nano Devices, Renmin University of China, Beijing, 100872 China

## Abstract

The relaxation of amorphous materials, i.e., aging, would largely endanger their performances in service. Here we report a mechanical relaxation-to-rejuvenation transition of a Zr_35_Ti_30_Be_27.5_Cu_7.5_ bulk metallic glass (BMG) in elastostatic compression at ambient temperature, thus provide an accessible way to tailor the mechanical properties of amorphous materials. To unravel the structural evolution underlying the observed transition, atomistic simulations parallel with the experimental tests on a typical model glass system Zr_60_Cu_40_ were performed, which successfully reproduced and thus upheld the experimentally observed mechanical relaxation-to-rejuvenation transition. The variations of coordination number and atomic volume during the transition are evaluated to indicate a de-mixing tendency of the constituent atoms in the rejuvenation stage. This de-mixing tendency largely explains the difference between mechanical rejuvenation and thermal rejuvenation and reveals a competitive relationship between activation enthalpy and activation entropy in the stress-driven temperature-assisted atomic dynamics of BMG, such as diffusion and plastic deformation etc.

## Introduction

The relaxation of amorphous materials, namely aging, a salient feature of these out-of-equilibrium systems^1, 2^ is a main detriment to their service reliability. Lately, it has been reported that via cyclic cryogenic process, “rejuvenation” of the amorphous structure, in the opposite direction to relaxation, can be achieved, leading to an enhancement of the room-temperature plasticity of bulk metallic glasses (BMGs)^3^. On the other hand, controlled structural rejuvenation via a tailored heating-and-quenching process has also been carried out to tune the deformability of BMG^4^. As a matter of fact, it has been revealed that stress and temperature are equivalent on the dynamics of BMG^5, 6^, i.e., the stress-temperature scaling, which indicates that the yield of BMG can be regarded as a stress-driven glass transition. Therefore, similar to the thermal rejuvenation revealed in BMG, stress-induced rejuvenation of the amorphous structure, i.e., mechanical rejuvenation, could be also achievable.

Actually, mechanical rejuvenation^7, 8^ has been observed as structural disordering inside shear bands in the plastic deformation of BMG^9, 10^ or in their supercooled liquids^11^, due to shear dilatation in the deformation of amorphous structure. Because of the susceptibility to shear banding after yield^12^, mechanical process before yield, i.e., elastostatic compression^13, 14^, is more preferred and has been used to optimize the mechanical properties of BMG. Although several Zr(Cu)-based BMGs shown elastostatic compressive stress-induced softening (i.e., rejuvenation)^15, 16^, elastostatic compressive stress-induced hardening (i.e., relaxation) of similar Zr(Cu)-based BMGs has also been observed^17, 18^. These results remind one of the thermal effect of temperature on the structure of BMGs, where sub-*T*_*g*_ annealing (*T*_*g*_ is the glass transition temperature) would lead to the annihilation of free volume^19^, i.e., relaxation, while sup-*T*_*g*_ annealing (i.e., annealing at temperature above *T*_*g*_) would lead to the creation of free volume, i.e., rejuvenation. Therefore, it is instinctive to anticipate a similar effect of elastostatic compressive stress on the structure of BMGs. Comparing the yield stress *σ*_*y*_ to *T*_*g*_, elastostatic compression at stresses below *σ*_*y*_ may lead to the annihilation of free volume and the relaxation of metallic glasses^18^, while elastostatic compression at stresses above *σ*_*y*_ may lead to the creation of free volume and the rejuvenation of metallic glasses^10^. Actually, it has been proposed in simulations that mechanical rejuvenation would not occur at stresses below yield stress, but only accelerated relaxation could be achieved^20–22^. These deductions suggest a possible existence of a mechanical relaxation-to-rejuvenation transition driven by stress^23, 24^. The existence of such a mechanical relaxation-to-rejuvenation transition driven by stress would largely explain the results that both softening and hardening are observed in the elastostatic compression of BMGs. If there were a mechanical relaxation-to-rejuvenation transition with increasing elastostatic stress, as the elastostatic stress approaching the yield stress, mechanical relaxation (i.e., stress-induced hardening) will finally transit into mechanical rejuvenation (i.e., stress-induced softening) in accordance with the stress-driven glass transition on the stress–temperature scaling diagram^6^. However, the critical stress of the transition from mechanical relaxation to mechanical rejuvenation may vary for different BMGs depending on the topological and chemical aspects of their amorphous structures^13, 14^. Therefore, at similar elastostatic stress levels, the BMGs with a lower critical stress would exhibit stress-induced softening, while the BMGs with a higher critical stress would exhibit stress-induced hardening. This is probably why both stress-induced softening and stress-induced hardening are found in different works^14, 18^.

On the other hand, unlike thermal rejuvenation, it is argued that mechanical deformation does not literally rejuvenate a glassy material^20^. Compared to the atomic configurations which can be obtained in thermal rejuvenation, only certain atomic configurations can be achieved in mechanical rejuvenation. Therefore, currently, whether such a mechanical relaxation-to-rejuvenation transition exists and what cause the fundamental differences between thermal rejuvenation and mechanical rejuvenation are two important issues in the study of rejuvenation of metallic glasses.

In this work, combined with molecular dynamics (MD) simulations, a transition from relaxation to rejuvenation in a typical Zr-TM(transition metal)-based BMG subjected to elastostatic compression at increasing stress levels is observed in both experiments and MD simulations. Thermal analysis, uniaxial compression, and density measurements on the post-elastostatic compressed samples are systematically conducted to confirm the observations in experiments. The underlying structural evolution in the mechanical relaxation-to-rejuvenation transition is revealed based on the variations of local atomic symmetry, coordination numbers, and atomic volumes in MD simulations. Finally, the atomistic mechanism of the mechanical relaxation-to-rejuvenation transition and the differences between thermal rejuvenation and mechanical rejuvenation are elucidated.

## Results and Discussion

### Mechanical relaxation-to-rejuvenation transition

**Figure 1:**
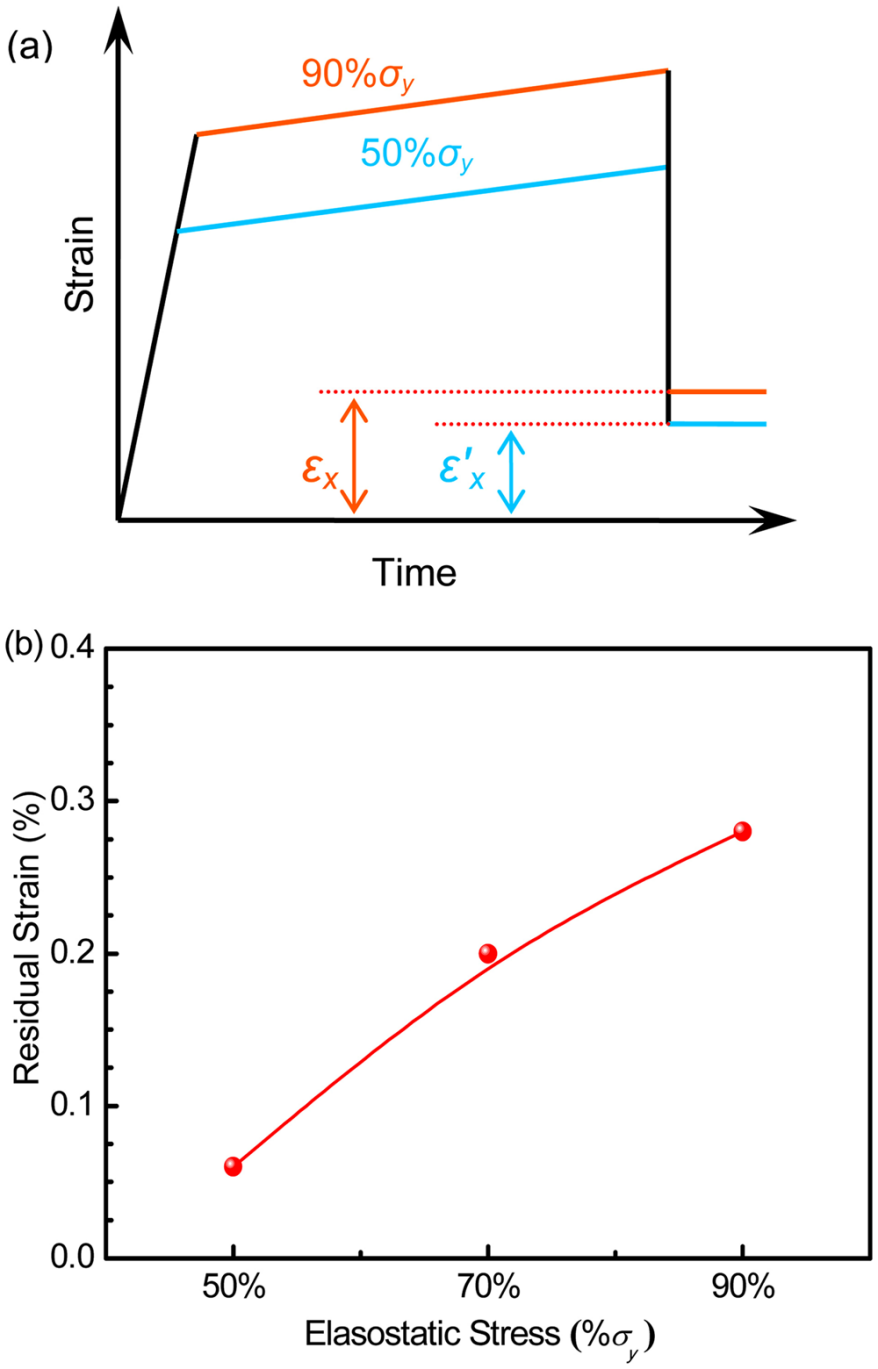
The elastostatic compression of Zr_35_Ti_30_Be_27.5_Cu_7.5_ bulk metallic glass at different stress values (The specimen is first loaded to a stress level of 50% and 90% of the yield stress *σ*_*y*_, respectively, and the stress was held for 48 h before unloading). (**a**) Strain evolution, *ε*_*x*_ and ${\varepsilon }_{x}^{^{\prime} }$ are the residual plastic strain after elastostatic compression at 90% *σ*_*y*_ and 50% *σ*_*y*_, respectively; (**b**) residual strain.

Figure 1(a) shows the strain evolution of the Zr_35_Ti_30_Be_27.5_Cu_7.5_ BMG in the elastostatic compression at different stress levels, during which the specimen was first loaded with a strain rate of 5 × 10^−4^ s^−1^ to a stress level of 50% and 90% of the yield stress *σ*_*y*_, respectively and then held there for a period of 48 h before unloading. It can be seen that during the elastostatic compression, even in the nominally elastic regime (*σ* < *σ*_*y*_) of the BMG, the strain increases slowly but monotonously with the extended loading time. After unloading, similar to what has been observed in previous studies^15, 16^, residual strains at the two stress levels applied can be detected on the post-elastostatic compressed specimens as shown in Fig. 1(b). In addition, the residual strain *ε*_*x*_ increases with increasing elastostatic compressive stress. However, the surface of all the tested specimens shows no traces of shear bands (not shown), indicating that the residual strain comes from homogenous deformation of the BMG specimen.

**Figure 2:**
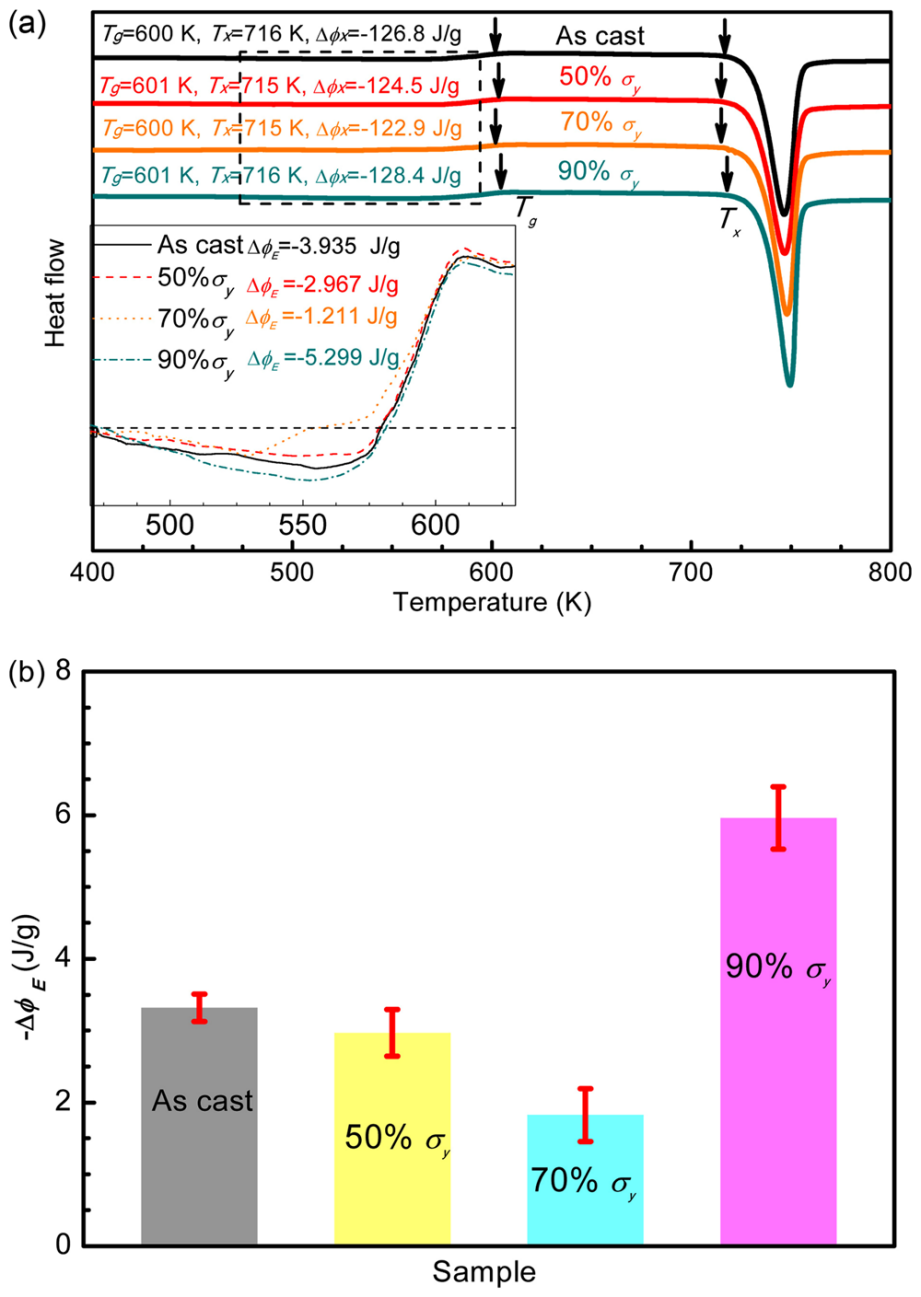
Thermograms of the as-cast and the post-elastostatic compressed specimens of Zr_35_Ti_30_Be_27.5_Cu_7.5_ bulk metallic glass at different elastostatic stresses for 48 h. (**a**) Differential scanning calorimetry curves, inset shows the closeup of the relaxation enthalpy for different specimens, i.e., the region highlighted by the rectangle; (**b**) relaxation enthalpy.

Figure 2(a) shows the DSC thermograms of the as-cast and the post-elastostatic compressed specimens of Zr_35_Ti_30_Be_27.5_Cu_7.5_ BMG. The characteristic glass transition temperature (*T*_*g*_), the crystallization temperature (*T*_*x*_) and the crystallization enthalpy (Δ*ϕ*_*X*_) for all the tested specimens are given in the same panel. It can be seen that the post-compressed specimens do not show appreciable changes in crystallization temperature *T*_*x*_ and crystallization enthalpy Δ*ϕ*_*X*_ as compared to the as-cast one, suggesting that the specimens remain in the amorphous state after elastostatic compression. The inset of Fig. 2(a) is the closeup of the region highlighted by the rectangle, where the individual thermograms are superimposed. The thermograms show a broad and shallow exothermic peak before glass transition, which represents the relaxation of excess enthalpy frozen in the BMG (also called relaxation enthalpy Δ*ϕ*_*E*_) during the rapid cooling process in copper mold casting^25^. Upon glass transition, an endothermic peak, which corresponds to the increase of enthalpy, i.e., rejuvenation, can be observed before the thermograms reach a plateau^26^. Namely, with increasing temperature, the specimens of Zr_35_Ti_30_Be_27.5_Cu_7.5_ BMG show a thermal induced relaxation-to-rejuvenation transition. Changes in Δ*ϕ*_*E*_ have been used to characterize the degree of aging and rejuvenation of metallic glasses^3^. Figure 2(b) shows the relaxation enthalpy Δ*ϕ*_*E*_ of the as-cast specimen and the post-elastostatic compressed specimens. It can be seen that, with respect to the as-cast specimen (Δ*ϕ*_*E*_ = 3.935 J/g), the relaxation enthalpy Δ*ϕ*_*E*_ of the post-elastostatic compressed specimen reduces to 2.967 J/g and 1.211 J/g after elastostatic compression at a stress of 50% and 70% *σ*_*y*_, respectively, but increases to 5.299 J/g after elastostatic compression at a stress of 90% *σ*_*y*_. Similar to the thermograms where the specimens show a relaxation-to-rejuvenation transition with increasing temperature, the relaxation enthalpy Δ*ϕ*_*E*_ of the post-elastostatic compressed specimens clearly indicates a transition from mechanical relaxation at lower stress level to mechanical rejuvenation at higher stress level with increasing elastostatic compressive stress.

**Figure 3:**
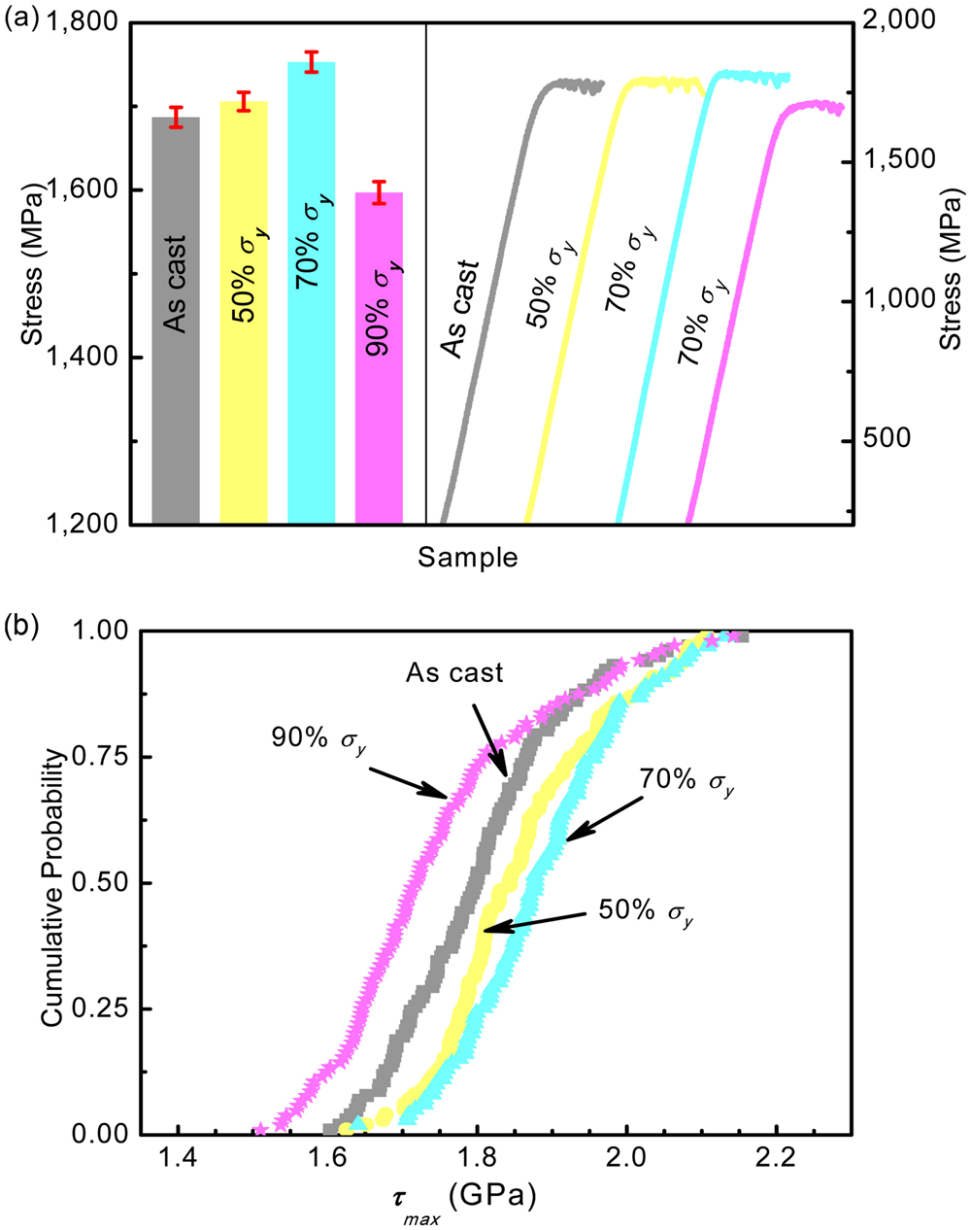
Yield stress of the as-cast and the post-elastostatic compressed specimens of Zr_35_Ti_30_Be_27.5_Cu_7.5_ bulk metallic glass at different elastostatic stresses for 48 h. (**a**) Yield stress and stress-strain curves; (**b**) the shear stress *τ*_max_ at the burst of the first pop-in event in nanoindentation.

It is generally accepted that relaxation would increase the strength of metallic glasses, but rejuvenation would contrarily reduce the strength of metallic glasses^27^. Consequently, quasi-static compression tests on the post-elastostatic compressed specimens of Zr_35_Ti_30_Be_27.5_Cu_7.5_ BMG were carried out and the corresponding stress-strain curves are collected in Fig. 3(a). It can be seen that, compared to the as-cast specimen, the yield stress increases for the specimens compressed at the stress level of 50% *σ*_*y*_ and 70% *σ*_*y*_, but decreases for the specimen compressed at the stress level of 90% *σ*_*y*_. For example, the yield stress of the BMG is about 1706 MPa and 1753MPa after elastostatic compression at 50% *σ*_*y*_ and 70% *σ*_*y*_, respectively, which is about 1.13% and 3.91% increase of the yield stress as compared to as-cast specimen (1687MPa). However, the yield stress of the post-elastostatic compressed specimens at a stress level of 90% *σ*_*y*_ decreases to 1597 MPa, which is about 5.33% of reduction of the yield stress of the as-cast specimen. As an extra evidence, Fig. 3(b) shows the shear stress *τ*_max_ determined by the burst of the first pop-in event in nanoindentation^18^ of the as cast and post-elastostatic compressed specimens of Zr_35_Ti_30_Be_27.5_Cu_7.5_ BMG, which indicates the nucleation of the first shear band corresponding to the beginning of plastic deformation. It can be seen that the distribution of the shear stress *τ*_max_ shifts to higher stress values after elastostatic compression at 50% *σ*_*y*_ and 70% *σ*_*y*_, but to lower stress values after elastostatic compression at 90% *σ*_*y*_. This phenomenon is consistent with the compression results in Fig. 3(a). Therefore, the hardening (below 70% *σ*_*y*_) to softening (above 90% *σ*_*y*_) transition of the yield stress of the post-elastostatic compressed specimens with increasing elastostatic stress supports the mechanical relaxation-to-rejuvenation transition observed in Fig. 2.

**Figure 4:**
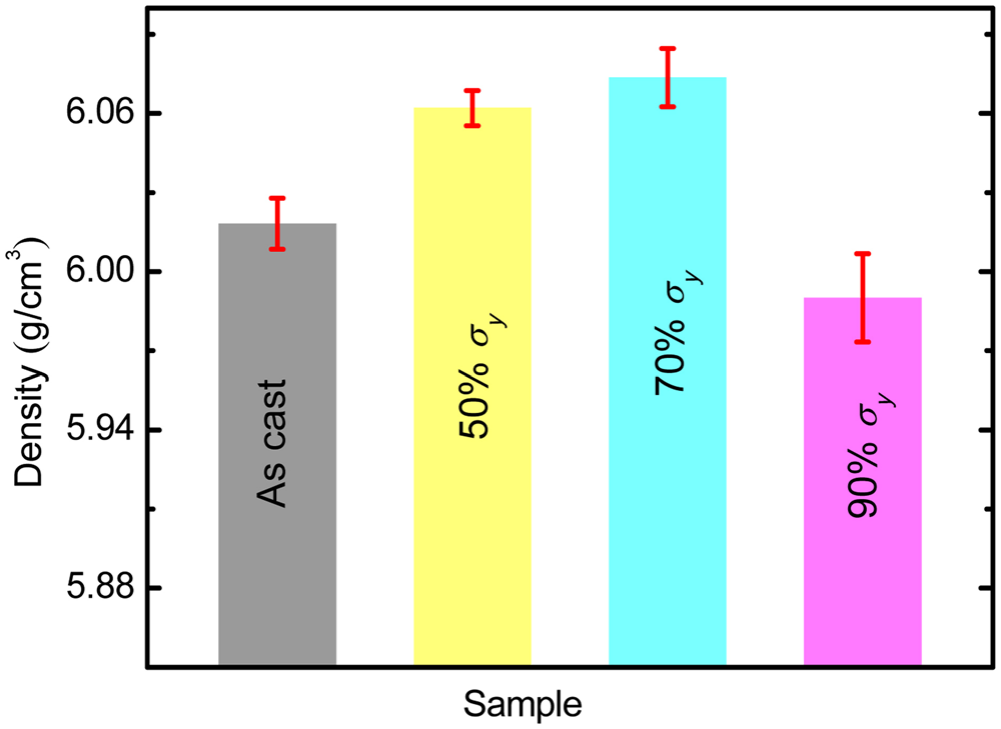
The density of the as-cast and the post-elastostatic compressed specimens of Zr_35_Ti_30_Be_27.5_Cu_7.5_ bulk metallic glass at different elastostatic stresses for 48 h.

Because the change in relaxation enthalpy Δ*ϕ*_*E*_ on thermograms is directly related to the change of free volume in metallic glasses^3^, the density of the post-elastostatic compressed specimens of Zr_35_Ti_30_Be_27.5_Cu_7.5_ BMG were also measured and compared to the as-cast specimen, as shown in Fig. 4. It can also be seen that the density of the specimens first increases and then decreases with increasing elastostatic compressive stress. For instance, the densities of the post-elastostatic compressed specimens at stress levels of 50% *σ*_*y*_ and 70% *σ*_*y*_ are 6.062 g/cm^3^ and 6.074 g/cm^3^, respectively, which are 0.73% and 0.92% higher than that of the as-cast specimen. However, the density of the post-elastostatic compressed specimen at 90% *σ*_*y*_ reduced to 5.990 g/cm^3^, which is 0.47% lower than that of the as-cast specimen. Therefore, in consistency with the variation of relaxation enthalpy Δ*ϕ*_*E*_ shown in Fig. 2, the density measurements also suggest that the free volume in the BMG was annihilated at the loading stress of 50% *σ*_*y*_ and 70% *σ*_*y*_, but was created at the loading stress of 90% *σ*_*y*_, indicating again a mechanical relaxation-to-rejuvenation transition in the BMG.

**Figure 5:**
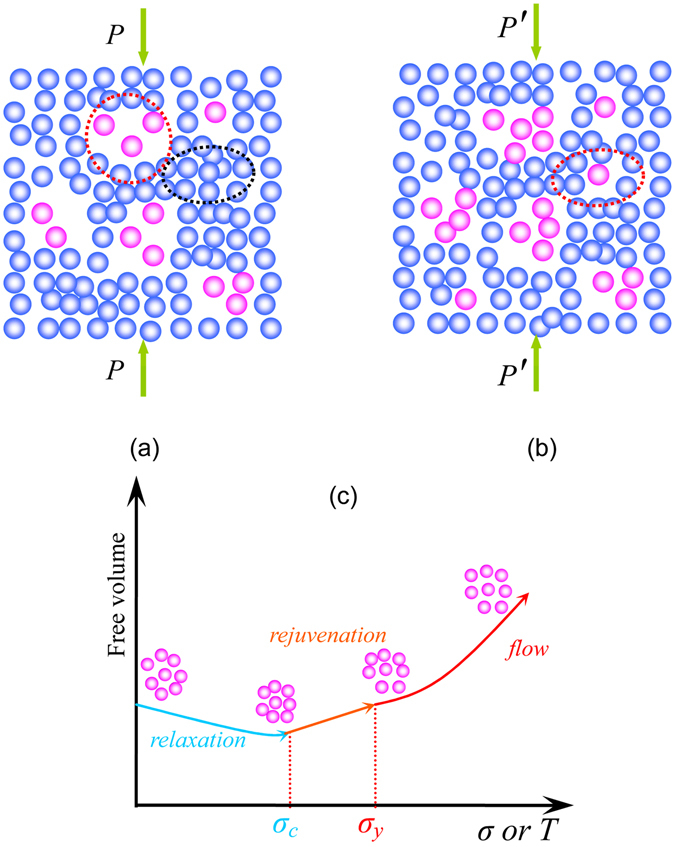
Schematic of the atomistic mechanism in the relaxation-to-rejuvenation transition and the existence of loosely packed regions and densely packed regions in bulk metallic glasses, i.e., the positive free volume sites and the negative free volume sites. (**a**) Under the effect of stress *P* which is below yield stress, stress-driven coalescence of the loosely packed regions and the densely packed regions would contribute to the homogeneous deformation of metallic glasses at ambient temperature; (**b**) Under the effect of stress *P*′ which is above yield stress, shear dilation would accompany the homogeneous deformation of metallic glasses at ambient temperature. (**c**) Schematic of the mechanical relaxation-to-rejuvenation transition: at stresses below a critical stress *σ*_*c*_, the effect of stress would accelerate aging, i.e. mechanical relaxation, while at stress above *σ*_*c*_, the effect of stress would rejuvenate the amorphous structure. At stresses above *σ*_*y*_, plastic flow starts.

It has already been explained that the room temperature homogenous plastic deformation of BMGs under elastostatic compression could originate from volume contraction, i.e., mechanical relaxation^18^. For the structural heterogeneities inherited from the supercooled liquid^28^, BMGs are proposed to involve both loosely packed and densely packed regions^29, 30^. As illustrated in Fig. 5(a), upon loading at stress *P* which is below yield stress, the coalescence of the loosely packed regions and the densely packed regions (i.e., the positive free volume sites and the negative free volume sites)^31, 32^ could occur, leading to the volume contraction and a permanent deformation, as indicated by the decreased relaxation enthalpy and increased density of the post-elastostatic compressed specimens as shown in Figs 2 and 4, respectively. As also being emphasized in the reported cyclic cryogenic rejuvenation^3^, It can be concluded that structural heterogeneity^33^ plays a crucial role in optimizing the performance of amorphous materials.

To understand the mechanical rejuvenation, it is important to recall the “work softening” of BMGs in plastic deformation due to the “shear-dilation” feature of amorphous structure^12, 34^, as the elastostatic compressive stress approaches the yield stress. Shear dilation have been confirmed in the deformation of a number of amorphous systems, such as BMGs and colloids^35, 36^. Once the applied stress is above yield stress, shear dilation would lead to the creation of free volume, i.e., structure rejuvenation of BMGs, as illustrated in Fig. 5(b). According to the results above, it can be seen that, at stresses below a critical stress *σ*_*c*_ which is approximately 70% *σ*_*y*_, the effect of stress leads to the relaxation of BMG, i.e. aging, while at stresses above *σ*_*c*_, the effect of stress leads to the rejuvenation of BMG. However, the critical stress *σ*_*c*_ is not equal to the yield stress *σ*_*y*_. Noting the ambiguous definition of *T*_*g*_^37^, the relationship between the critical stress *σ*_*c*_ and the yield stress *σ*_*y*_ is worth further investigation to understand the complex yield behavior of BMGs^10^. Consequently, as shown in Fig. 5(c), a mechanical relaxation-to-rejuvenation transition driven by the stress can be rationalized, just like the thermal glass transition on the thermograms of metallic glasses heated from room temperature to the supercooled liquid region. However, the atomistic mechanism of structural rejuvenation is still under debate^7^.

### The underlying structural evolution

To understand the mechanical relaxation-to-rejuvenation transition from a structural point of view, molecular dynamics (MD) simulations were performed on a model system of Zr_60_Cu_40_ metallic glass to examine the structural evolution under elastostatic compression at various stress levels.

**Figure 6:**
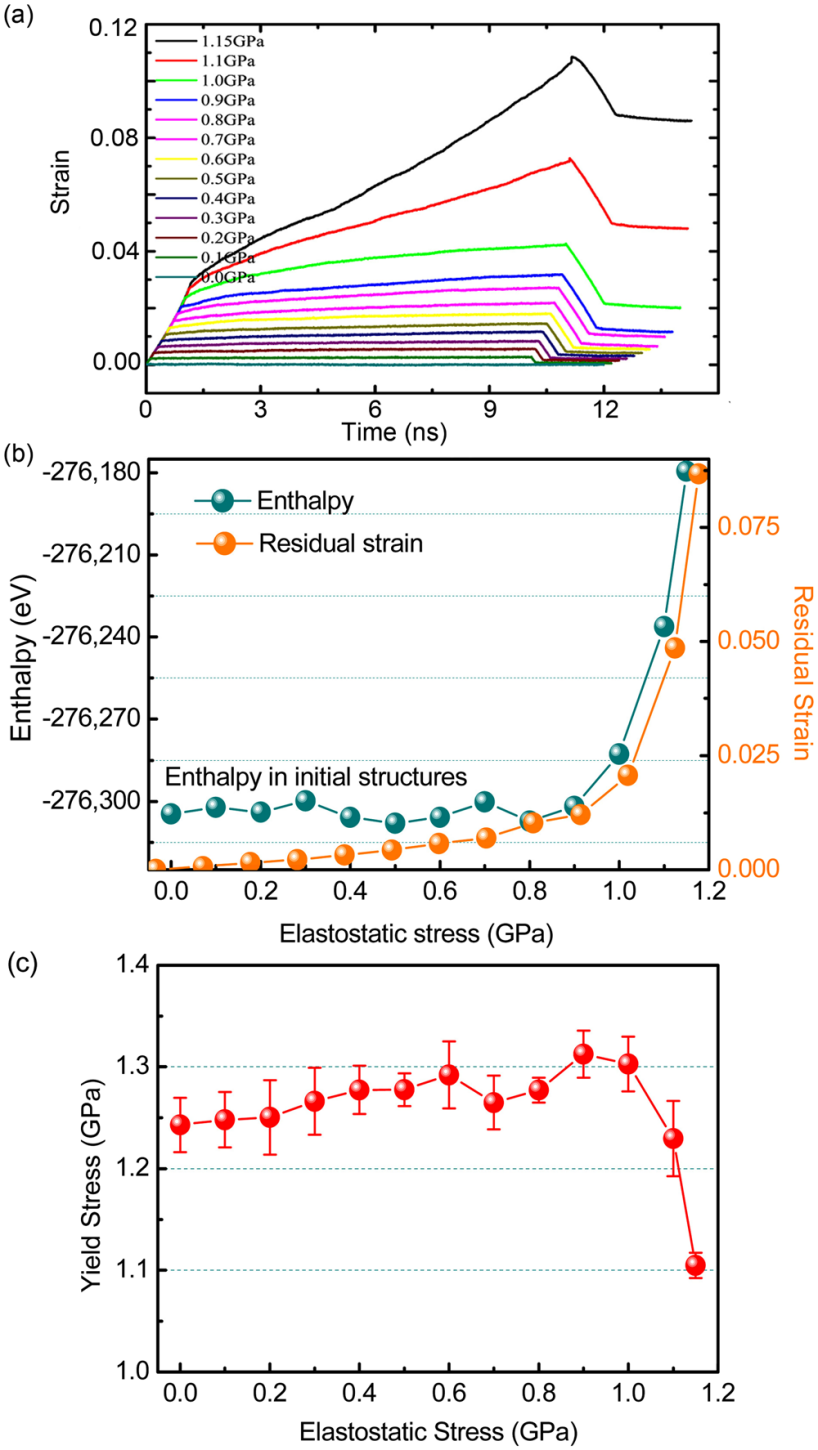
Results from molecular dynamics (MD) simulations of the elastostatic compression process of Zr_60_Cu_40_ metallic glass samples at different elastostatic stresses. (**a**) Strain evolution; (**b**) enthalpy and residual strain; (**c**) yield stress.

Figure 6(a) shows the strain evolution of Zr_60_Cu_40_ metallic glasses during elastostatic compression at different stress levels. The samples were first compressed with a loading rate of 10^−5^/ps. After the target stress was reached, elastostatic compression was followed for 10 ns, and then the samples were unloaded with an unloading rate of 10^−5^/ps and relaxed further for 2 ns. It is clearly seen that the behavior of strain *vs*. time is similar to the experimental results shown in Fig. 1(a), indicating that MD simulations are consistent with the experiments. As shown in Fig. 6(b), the residual strain after unloading increases monotonously with increasing elastostatic compressive stress, which is also consistent with the experimental results shown in Fig. 1(b). As the stress is larger than 0.9 GPa and close to the yield stress (*σ*_*y*_~1.25 GPa), the residual strain increases abruptly. We also calculated the enthalpy of the post-elastostatic compressed samples. As shown in Fig. 6(b), the enthalpies in the post-elastostatic compressed samples are lower than that in the sample before elastostatic compression test, as the stress is smaller than 0.9 GPa. However, in the cases of elastostatic compressive stress larger than 0.9 GPa, the enthalpy is getting higher than that of the sample before elastostatic compression test. This is again consistent with the experimental results shown in Fig. 2(b). This result indicates that the glassy sample relaxed to a lower energy state after the elastostatic compression test at stresses smaller than 0.9 GPa, while the sample is driven to higher energy states and the rejuvenation occurs as the stress is larger than 0.9 GPa. To make a further comparison with the experimental results, as shown in Fig. 6(c), the yield stress of the post-elastostatic compressed samples increases with increasing the elastostatic compressive stress before 0.9 GPa, and subsequently decreases with the elastostatic compressive stress when it is larger than 0.9 GPa. Thus, a hardening (from 0 to 0.9 GPa) to softening (from 0.9 GPa to above) transition is clearly displayed in Fig. 6(c). A critical stress *σ*_*c*_ can be determined as 0.9 GPa for the model system Zr_60_Cu_40_. Therefore, the MD simulations are consistent with the experiments shown in Fig. 3 and support the mechanical relaxation-to-rejuvenation transition in metallic glasses.

**Figure 7:**
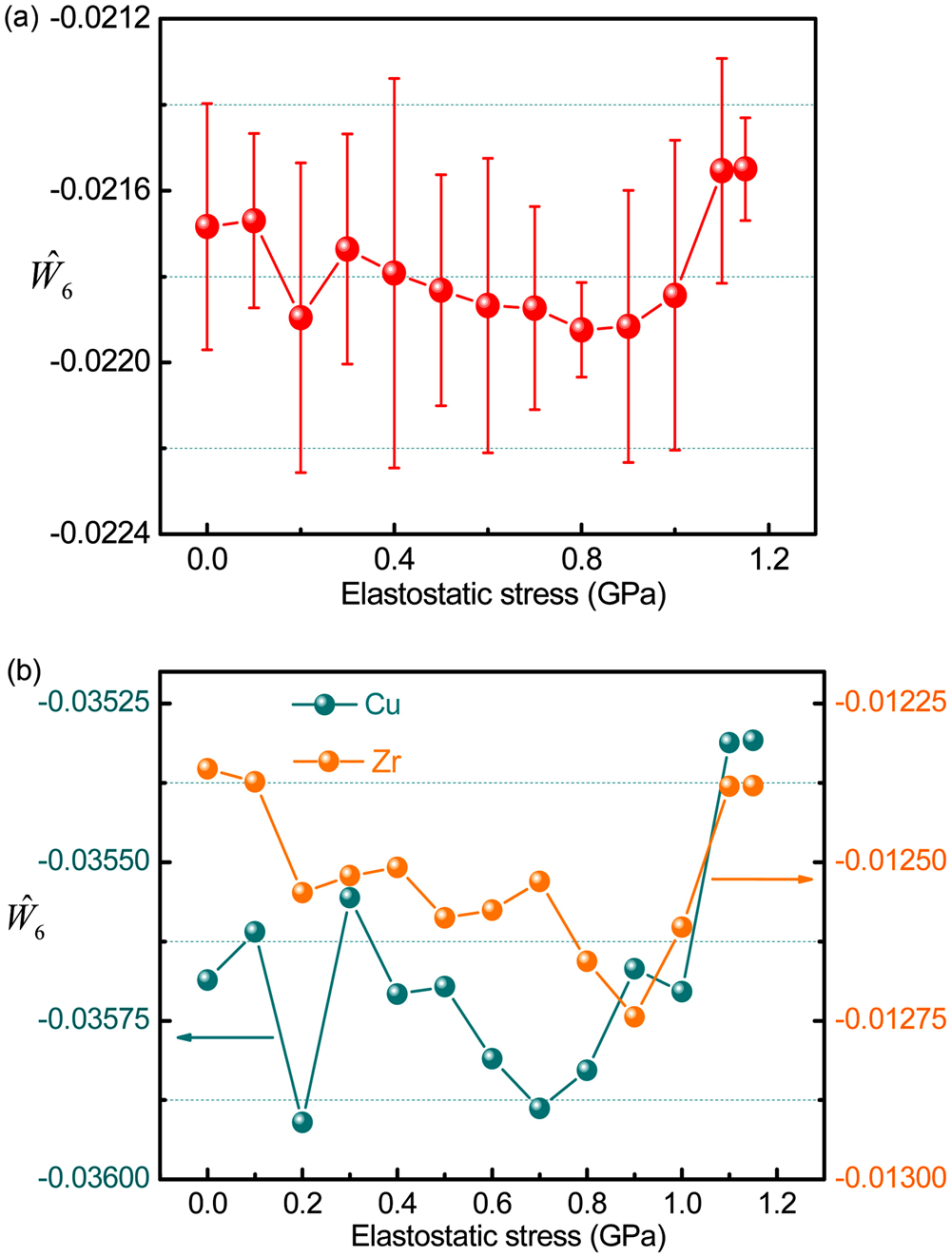
The evolution of bond-orientation order parameter in the post-elastostatic compressed Zr_60_Cu_40_ metallic glass samples with increasing elastostatic stress. (**a**) The averaged bond-orientation order parameter; (**b**) around Cu and Zr atoms.

For the non-affine nature of the deformation in metallic glasses, the evolution of the local atomic symmetry of the model Zr_60_Cu_40_ system would be very important to confirm the mechanical relaxation-to-rejuvenation transition. The local atomic symmetry can be characterized in terms of the bond-orientation order (BOO) parameter^38^. To quantify the bond-orientation symmetry around a central atom, BOO is defined according to the spherical harmonics as follows:

1 $${q}_{lm}({\vec{r}}_{i})=\frac{1}{{N}_{i}}\sum _{j=1}^{{N}_{i}}Y{}_{lm}(\theta ({\vec{r}}_{ij}),\,\varphi ({\vec{r}}_{ij}))$$

where *Y*_*lm*_ is spherical harmonics, and *N*_*i*_ is the coordination number of atom *i*. ${\vec{r}}_{i}$ is the position vector; $\theta ({\vec{r}}_{ij})$ and $\varphi ({\vec{r}}_{ij})$ are the polar angle of the bond ${\vec{r}}_{ij}$, respectively. The rotational invariants can thus be defined as:

2 $${Q}_{l}=\sqrt{\frac{4\pi }{2l+1}\sum _{m=-l}^{l}{|{q}_{lm}|}^{2}}$$

and

3 $${W}_{l}=\sum _{{m}_{1}+{m}_{2}+{m}_{3}=0}(\begin{array}{ccc}l & l & l\\ {m}_{1} & {m}_{2} & {m}_{3}\end{array}){q}_{l{m}_{1}}{q}_{l{m}_{2}}{q}_{l{m}_{3}},$$

where $$(\begin{array}{ccc}l & l & l\\ {m}_{1} & {m}_{2} & {m}_{3}\end{array})$$ is the Wigner 3-j symbol. The normalized parameter ${\hat{W}}_{l}={W}_{l}/{(\sum _{m=-l}^{l}{|{q}_{lm}|}^{2})}^{3/2}$ is defined to describe the different orientational symmetries and often used to evaluate the BOO and to differentiate the various local environments in dynamic crossover phenomena, such as glass transition and liquid-liquid phase transition in metallic glass-forming liquids^39, 40^. Particularly, ${\hat{W}}_{6}$ values of face-centered cubic (fcc), hexagonal close-packed (hcp), and icosahedron order local environments are −0.01316, −0.01244, and −0.16975, respectively. The value of ${\hat{W}}_{6}$ becomes more negative if the system has a stronger icosahedron-order-like local environment, i.e., more five-fold local symmetry. Therefore, ${\hat{W}}_{6}$ is a sensitive measure to characterize the evolution of five-fold local symmetry of local environments^41^ and adopted in the present study. Figure 7(a) shows the variation of ${\hat{W}}_{6}$ with increasing elastostatic compressive stress. It is clearly seen that ${\hat{W}}_{6}$ becomes more negative with increasing elastostatic compressive stress when the stress is smaller than 0.9 GPa, but becomes less negative as the stress is larger than 0.9 GPa. The critical stress *σ*_*c*_ for the model system Zr_60_Cu_40_ can also be determined as 0.9 GPa. This indicates that the atomic structures exhibit more five-fold symmetry at stresses below 0.9 GPa, while exhibit less five-fold symmetry at stresses above 0.9 GPa. Similar behaviors are also observed in the variation of ${\hat{W}}_{6}$ around Cu and Zr atoms, as shown in Fig. 7(b), indicating that the local atomic symmetry around both Cu and Zr atoms varies similarly with increasing elastostatic compressive stress. Because that the local atomic environments are prone to the configurations with more five-fold local symmetry in relaxation, while change to the configurations with less five-fold local symmetry in rejuvenation^41^, these local environments variations strongly confirm the mechanical relaxation-to-rejuvenation transition in Zr_60_Cu_40_ metallic glass.

**Figure 8:**
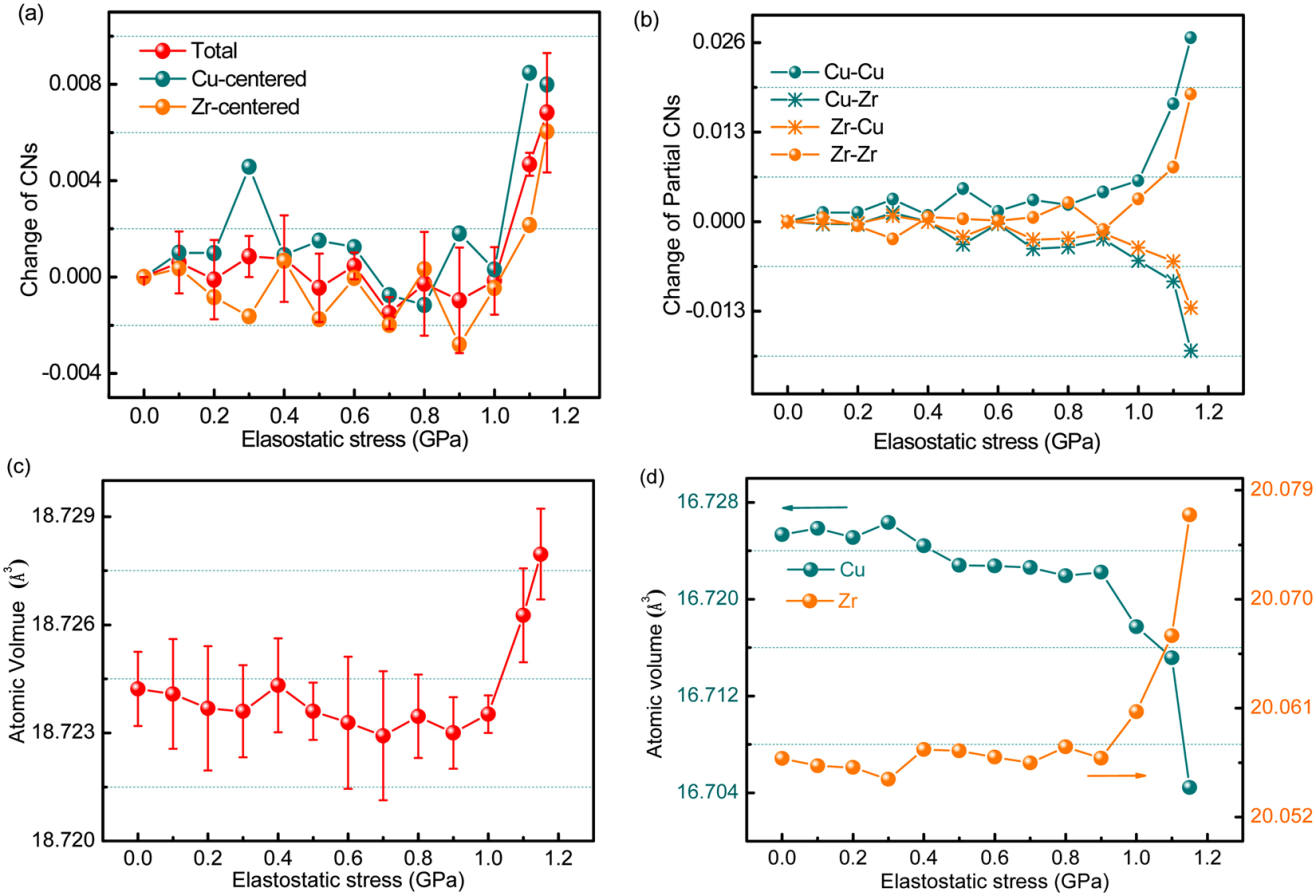
The variation of coordination number and atomic volume with respect to the initial Zr_60_Cu_40_ metallic glass samples at increasing elastostatic stress. (**a**) The change of the coordination numbers of total atoms, Cu and Zr atoms, respectively; (**b**) the change of the number of Cu and Zr atoms around Cu and Zr; (**c**) the variation of the averaged atomic volumes, and (**d**) the variation of atomic volumes of Cu and Zr atoms.

To reveal the evolution of the atomic rearrangements with increasing elastostatic compressive stress, Fig. 8(a) shows the change of the total coordination numbers (CNs) of the Zr_60_Cu_40_ metallic glass samples as function of the elastostatic compression stress. Although the CNs show fluctuation with elastostatic compression stress, the tendency for the change is clear, i.e., CNs decreases (from 0 to 0.9 GPa) and then increases (from 0.9 GPa to above) with increasing stress. The partial CNs of Cu and Zr atoms also exhibit similar behaviors as shown in Fig. 8(a). The behavior of the change of CNs is consistent with the change of the yield stress of the Zr_60_Cu_40_ MG in MD simulations (Fig. 6(c)) and the change of the density of the Zr_35_Ti_30_Be_27.5_Cu_7.5_ BMG (Fig. 4). This consistency indicates that the evolution of the atom packing is in alignment with the mechanical relaxation-to-rejuvenation transition. We also analyzed the change of the number of Zr and Cu atoms in the nearest-neighbor shells of Zr and Cu atoms, respectively. As shown in Fig. 8(b), the number of Cu atoms around Cu and the number of Zr atoms around Zr increase with increasing stress and jump abruptly as stress is larger than 0.9 GPa, while the number of Cu atoms around Zr and the number of Zr atoms around Cu decreases as stress increases. This result indicates that Zr_60_Cu_40_ metallic glass exhibits a de-mixing tendency of the constituent atoms of Cu and Zr in the mechanical rejuvenation (from 0.9 GPa to above) process. This de-mixing tendency in rejuvenation can be interpreted based on the negative and positive free volume distribution in metallic glasses in the framework of atomic level stress model^31^ (see Fig. 5(a)). The negative free volume might aggregate around the Cu-Zr bond (0.27 nm) because of its relatively larger size mismatch and higher packing efficiency; while positive free volume might locate near Zr-Zr (0.32 nm) bond due to its relative lower packing efficiency^42^. Therefore, the dilation effect in the rejuvenation process may lead to the decreasing of Cu-Zr bonds and the increasing of Zr-Zr bonds. Figure 8(c) shows the variation of the atomic volume with increasing compressive stress. The atomic volume decreases with increasing stress up to 0.9 GPa, but increases when the stress is above 0.9 GPa. The behavior of the atomic volume is also consistent with the change in density of the post-elastostatic compressed BMG specimens measured in experiments (see Fig. 4). Figure 8(d) shows the variation of the atomic volume of Zr and Cu atoms with increasing stress, respectively. While the atomic volume of Zr atoms increases as the stress goes beyond 0.9 GPa, the atomic volume of Cu atoms decreases. This result is consistent with the de-mixing tendency between the Zr and Cu atoms indicated in Fig. 8(b), because that rejuvenation would result in the increase of Zr-Zr bonds and the decrease of Cu-Zr bonds as interpreted above, and consequently lead to the increase of the atomic volume of Zr atoms and the decrease of the atomic volume of Cu atoms. Therefore, it can be concluded that the evolution of the short range order structures may be responsible for the mechanical relaxation-to-rejuvenation transition.

### Significance of the de-mixing tendency

The de-mixing tendency of the constituent atoms in mechanical rejuvenation has also been detected experimentally in previous studies in a series of ZrCu-based metallic glasses, in which elastostatic compression leads to the break of Zr-Cu bonds and the formation of Zr-Zr bonds^43^. This de-mixing tendency could also be the reason for the elastic anisotropy induced by flow in metallic glasses^44^. It is suggested that, in mechanical rejuvenation, stress can change the shape of the potential energy landscape and drive the system into phase spaces that could not be reached in thermal rejuvenation^20–22^. It is important to note that thermal rejuvenation refers to the rejuvenation process caused by heating metallic glasses into the supercooled liquid region. Thus, the observed de-mixing tendency of Zr and Cu atoms may indicate the differences between mechanical rejuvenation and thermal rejuvenation, because heating may increase the entropy of the system, but not be likely to induce such a de-mixing tendency; nonetheless, external loading would introduce energy into the system via mechanical work and cause new favorite atomic configurations, i.e., a de-mixing tendency of the constituent atoms, as shown in Fig. 8(b). Specifically, the activation Gibbs free energy Δ*G* in the structural evolution of metallic glasses reads: Δ*G* = Δ*H* − *T*Δ*S* = Δ*Q* − *τ*Δ*V* − *T*Δ*S*, where Δ*Q* is the activation energy; Δ*H* is the activation enthalpy; Δ*S* is the activation entropy; Δ*V* is the activation volume; *T* is temperature. Based on the empirical enthalpy-entropy compensation rule^45^ which also prevails in the dynamics of metallic glasses^46^, for the high level of stress *τ* in mechanical rejuvenation, the activation enthalpy Δ*H* would dominate in Δ*G* and suppress the effect of activation entropy Δ*S* and drive the rejuvenated system into the identical energy minima as reported in ref. 20. Hence, the de-mixing tendency of the constituent atoms in mechanical rejuvenation is probably due to the competition between the activation enthalpy Δ*H* and the activation entropy Δ*S*. As to thermal rejuvenation, without mechanical stimulus, the activation entropy dominates the activation dynamics of the structural evolution process. Hence, as metallic glasses are heated into supercooled liquid region in thermal rejuvenation, the entropy of metallic glasses would increase and the amorphous structure would be more homogeneous and disorder, which would prevent the occurrence of such a de-mixing tendency. This de-mixing tendency largely explains the difference between mechanical rejuvenation and thermal rejuvenation and reveals a competitive relationship between activation enthalpy and activation entropy in the stress-driven temperature-assisted atomic dynamics of condensed matters, such as diffusion and plastic deformation^46^ etc. Moreover, for the equivalent effects of stress and temperature on the fluctuation-dominated dynamics of metallic glasses, the de-mixing tendency will also be important to understanding the dynamics in glass transition from atomic packing^47^.

## Conclusion

To summarize, a mechanical relaxation-to-rejuvenation transition in a Zr_35_Ti_30_Be_27.5_Cu_7.5_ metallic glass was clearly observed in elastostatic compression, as verified experimentally with differential scanning calorimetry, density measurements and uniaxial compression and confirmed theoretically with molecular dynamics (MD) simulations. The structural analyses based on MD simulations revealed that the observed mechanical relaxation-to-rejuvenation transition is associated with the change of local atomic symmetry in the metallic glass, where the relaxation process leads to the reduction of the bond-orientation order (BOO) ${\hat{W}}_{6}$ and enhancement of five-fold local symmetry, while the rejuvenation process results in the increase of ${\hat{W}}_{6}$ and weakness of five-fold local symmetry. The existence of mechanical relaxation supports the existence of structural heterogeneity in metallic glasses. The variation of the coordination numbers and the atomic volumes in mechanical rejuvenation reveals a de-mixing tendency of the constituent atoms. The de-mixing tendency indicates that the evolution of the short range order structures may be responsible for the mechanical relaxation-to-rejuvenation transition. Finally, the de-mixing tendency also suggests that the difference between thermal rejuvenation and mechanical rejuvenation might lie in the competition between the activation enthalpy and the activation entropy.

## Methods

Rods of Zr_35_Ti_30_Be_27.5_Cu_7.5_ bulk metallic glass (BMG) with a diameter of 3 mm were prepared via copper-mold casting. Specimens with an aspect ratio of 2:1 (length:diameter) were cut from the as-cast rods, of which both the ends were polished carefully to ensure the parallelism. Respectively, compressive stresses of 50%, 70% and 90% of *σ*_*y*_(*σ*_*y*_ = 1687 MPa, yield stress) were loaded at a strain rate of 5 × 10^−4^ s^−1^ on the specimens and maintained (i.e. elastostatic compression) for a period of 48 hours at room temperature (RT, ~298 K = 0.44*T*_*g*_) on an electromechanical testing machine. The enduring time of 48 hours is chosen based on our previous work^18^, after which the structural evolution of BMG would be approximately saturated. The structure change in specimens after the elastostatic loading was characterized with differential scanning calorimeter (DSC, TA Q-2000) at a heating rate of 20 K/min. A second run of each specimen under the identical condition was performed as the reference to calculate the relaxation enthalpy. Quasi-static compressive tests until failure were conducted at a strain rate of 5 × 10^−4^ s^−1^ to measure the yield stress of the as-cast and post-elastostatic compressed specimens. All tests are repeated at least 3–5 times to guarantee the data reliability. Density measurements of the post-elastostatic compressed specimens were carried out based on Archimedes’ method using a high precision balance with an accuracy of ±0.01 mg. At least 15 times of measurements were performed to ensure the data reliability.

Moreover, the elastostatic compression was carried out on a model system of Zr_60_Cu_40_ metallic glass in MD simulations with a realistic interatomic interaction potential^48^. The Zr_60_Cu_40_ sample containing 54000 atoms was modeled in a rectangular box (X:Y:Z = 1:2:1) with periodic boundary conditions. The sample was first melted and equilibrated at 2000 K for 2 ns in NPT (constant atom number, constant pressure, and constant temperature) ensemble, then cooled down to 300 K at a cooling rate of 1 K/ps to obtain the glassy state. The glass transition temperature is determined to be *T*_*g*_ = 808 K. To eliminate the deviation caused by test temperature, all the simulation tests are also performed at 0.44*T*_*g*_ = 355 K. Before the elastostatic compression test on the samples, we performed compression test on the samples with a typical loading rate of 10^−5^/ps and yield stresses of the samples were determined as about 1.25 GPa in the simulations. Thus, the simulations of the elastostatic compression tests were performed with the stress below the yield stress in the range of 0 GPa and 1.15 GPa at an interval of 0.1 GPa. In the elastostatic compression at 0 GPa, the metallic glass samples are subjected to purely thermal annealing. In the simulations, five independent metallic glass samples were prepared and the elastostatic compression tests with the same mechanical conditions were then performed for statistical analysis. In the above simulations, the time step used to integrate the equation of motion is chosen as 1 fs and the temperature was controlled using the Nose-Hoover thermostat.
